# Emergency rescue esophageal stenting through retrograde and antegrade rendezvous gastroscopy for an iatrogenic complicated esophageal perforation

**DOI:** 10.1055/a-2164-0490

**Published:** 2023-11-14

**Authors:** Yi Yang, Chuntao Liu, Chuyan Chen, Jie Yin, Xiaoye Liu, Peng Li

**Affiliations:** 1 Department of Gastroenterology, Capital Medical University Affiliated Beijing Friendship Hospital, Beijing, China; 2 Department of General Surgery, Capital Medical University Affiliated Beijing Friendship Hospital, Beijing, China

Endoscopic submucosal dissection (ESD) is the primary treatment for superficial esophageal cancer 1 . Post-ESD esophageal stenosis is commonly managed with endoscopic balloon dilation 2 . We present the case of a 76-year-old man with esophageal rupture during balloon dilation for post-ESD stenosis after early-stage esophageal cancer treatment. Despite prophylactic glucocorticoid and repeated endoscopic radial incisions, stenosis recurred.

Gastroscopy identified a pinhole-like stenosis 18 cm from the incisors ( Fig. 1 ). We planned endoscopic balloon dilation and subsequent self-expanding metal stent (SEMS) implantation. The contrast agent revealed a linear extension from the stenosis into the stomach. After multiple attempts, the guidewire crossed the stenosis, dilating it to 0.8 cm ( Fig. 2 ). Unfortunately, it mistakenly entered the mediastinum, causing perforation and contrast extravasation. Limited space precluded clip closure or suturing. SEMS implantation was deemed high risk due to uncertainty in accessing the true lumen. Endoscopic treatment was halted.

**Fig. 1:**
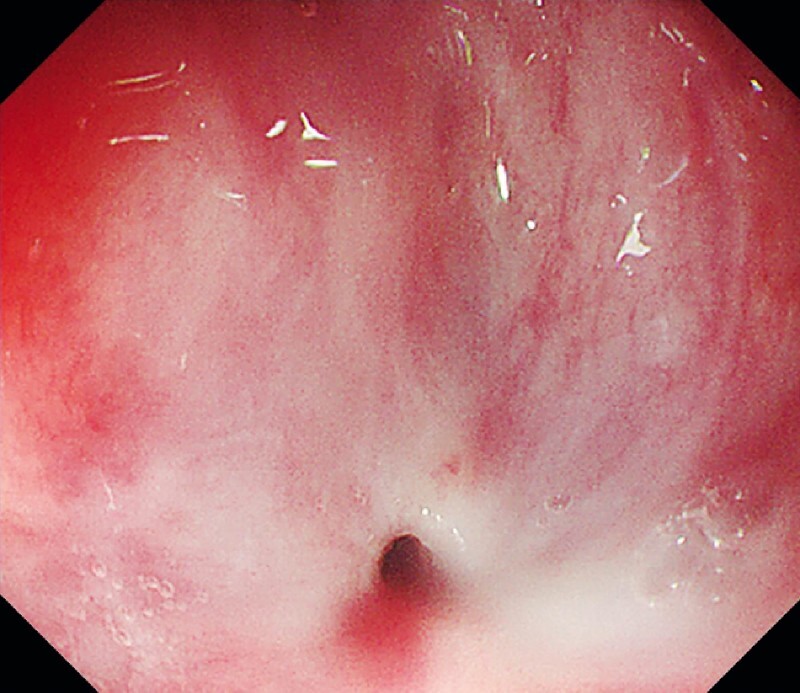
Circumferential stenosis in the upper esophagus.

**Fig. 2:**
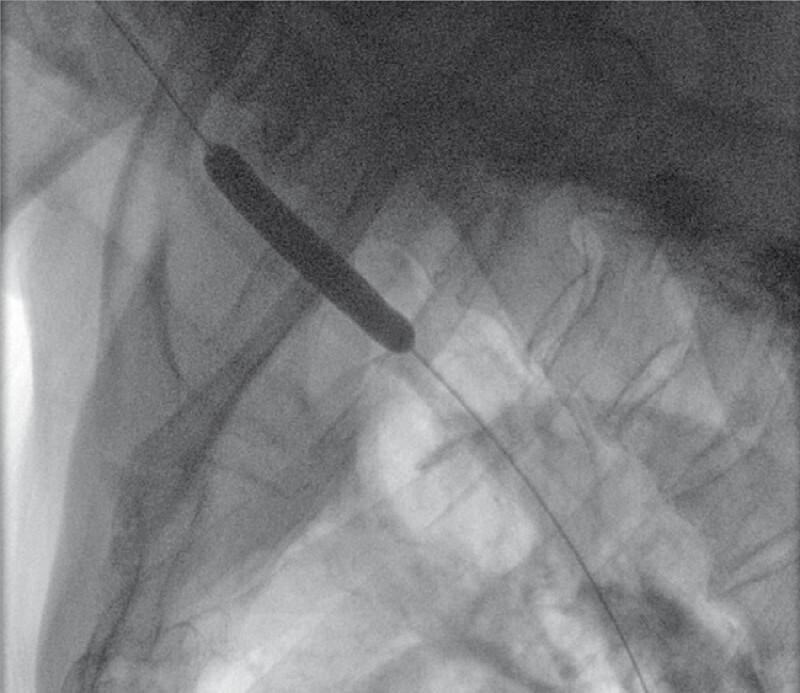
Balloon dilation at the false lumen.

The patient experienced progressive chest pain and pulse oxygen decline. Emergency computed tomography (CT) showed esophageal perforation with mediastinal gas accumulation ( Fig. 3 ), contrast leakage ( Fig. 4 ), and bilateral pneumonia. Cefoperazone/sulbactam therapy was started immediately.

**Fig. 3:**
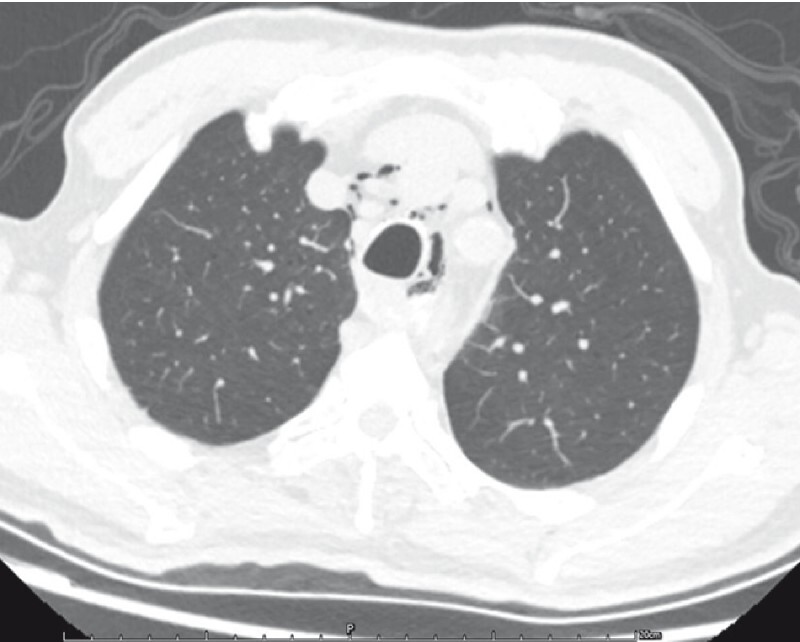
Esophageal perforation with gas beside the esophagus.

**Fig. 4:**
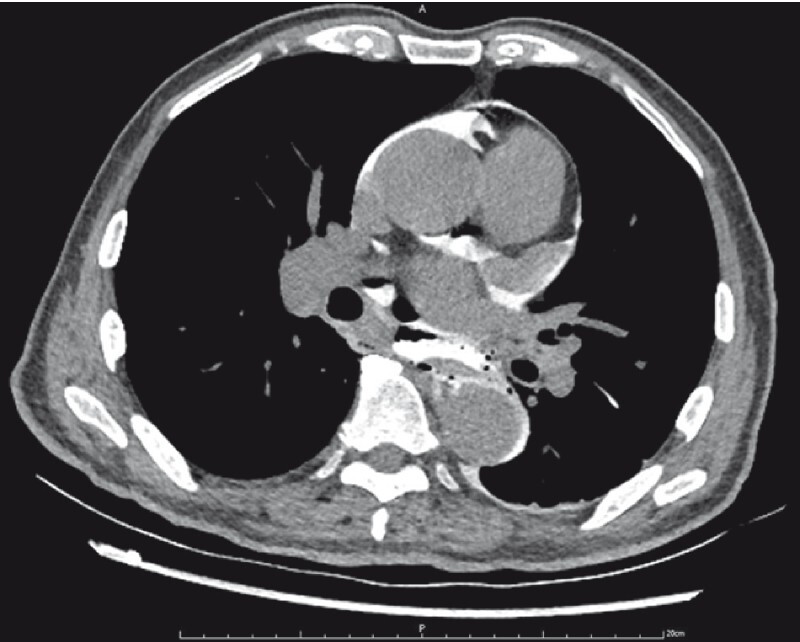
Contrast agent leakage into the pericardium.

A retrograde-antegrade rendezvous gastroscopy via surgical gastrostomy was performed for esophageal SEMS implantation. Surgeons assisted in creating a temporary gastrostomy for retrograde gastroscope access. A disposable gastroscope was used via the surgical gastrostomy to maintain sterility. An antegrade gastroscope approached the esophageal stricture orally. A guidewire traversed the stenosis via the retrograde gastroscope and was grasped with an endoscopic snare orally. A fully covered SEMS was placed anterogradely, covering the perforation and stenosis; both gastroscopic views confirmed the stent position. The stent was fixed with a clip to prevent migration ( Video 1 ).

**Video 1** Emergency rescue esophageal stenting through retrograde and antegrade rendezvous gastroscopy for an iatrogenic complicated esophageal perforation.


Infection control was achieved post-procedure, and the patient resumed a regular diet without stent intolerance. A 3-week follow-up chest CT scan revealed gas absorption and pneumonia improvement ( Fig. 5 ).

**Fig. 5:**
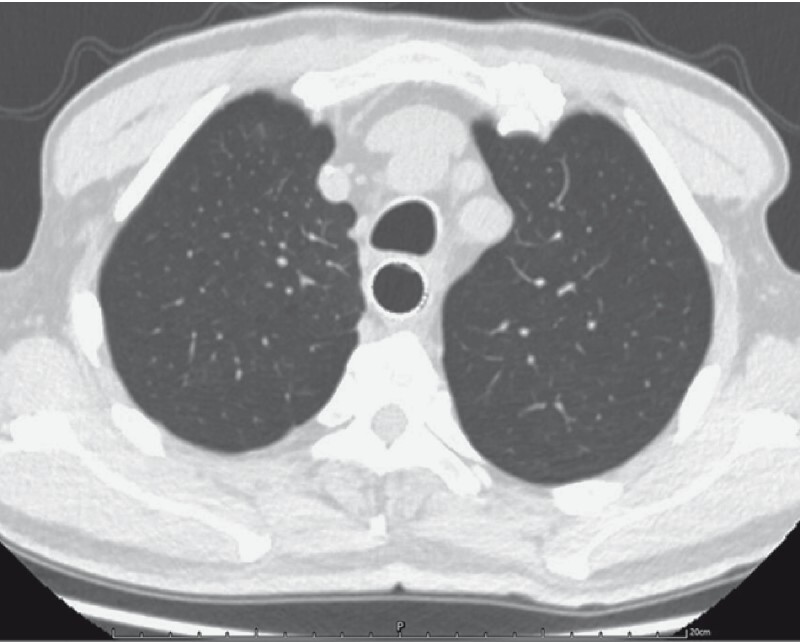
The previously observed gas in the mediastinum had been largely absorbed at the 3-week follow-up.

Retrograde-antegrade rendezvous gastroscopy for SEMS implantation offers a promising alternative rescue therapy for acute iatrogenic esophageal rupture, potentially avoiding more invasive surgery.

Endoscopy_UCTN_Code_CPL_1AH_2AG

## Notes

Endoscopy E-Videos

https://eref.thieme.de/e-videos
*E-Videos* is an open access online section of the journal *Endoscopy* , reporting on interesting cases and new techniques in gastroenterological endoscopy. All papers include a high-quality video and are published with a Creative Commons CC-BY license. Endoscopy E-Videos qualify for HINARI discounts and waivers and eligibility is automatically checked during the submission process. We grant 100% waivers to articles whose corresponding authors are based in Group A countries and 50% waivers to those who are based in Group B countries as classified by Research4Life (see: https://www.research4life.org/access/eligibility/ ). This section has its own submission website at https://mc.manuscriptcentral.com/e-videos
